# Description of lipase producing novel yeast species *Debaryomyces apis* f.a., sp. nov*.* and a modified pH indicator dye-based method for the screening of lipase producing microorganisms

**DOI:** 10.1038/s41598-023-38241-3

**Published:** 2023-07-21

**Authors:** Alka Kumari, Kanti N. Mihooliya, Debendra K. Sahoo, Mani S. Bhattacharyya, Gandham S. Prasad, Anil Kumar Pinnaka

**Affiliations:** 1 Microbial Type Culture Collection (MTCC), CSIR-Institute of Microbial Technology, Chandigarh, 160036 India; 2 Biochemical Engineering Research and Process Development Centre, CSIR-Institute of Microbial Technology, Chandigarh, 160036 India; 3 Present Address: Centre for Knowledge Culture and Innovation Studies, Technology Industrial Liaison and Entrepreneurship Unit, University of Hyderabad, Prof. C R Rao Road, Gachibowli, Hyderabad, Telangana 500046 India

**Keywords:** Biological techniques, Microbiology

## Abstract

Four yeast strains were isolated from the gut of stingless bee, collected in Churdhar, Himachal Pradesh, India. Physiological characterization, morphological examination, and sequence analysis of small subunit ribosomal RNA (18S rRNA) genes, internal transcribed spacer (ITS) region, and D1/D2 domain of the large subunit rRNA gene revealed that the four strains isolated from the gut of stingless bee belonged to the *Debaryomyces* clade. Strain CIG-23H^T^ showed sequence divergence of 7.5% from *Debaryomyces nepalensis* JCM 2095^T^, 7.8% from *Debaryomyces udenii* JCM 7855^T^, and *Debaryomyces coudertii* JCM 2387^T^ in the D1/D2 domain. In the ITS region sequences, strain CIG-23H^T^ showed a 15% sequence divergence from *Debaryomyces nepalensis* JCM 2095^T^ and *Debaryomyces coudertii* JCM 2387^T^. In 18S rRNA gene sequence, the strain CIG-23H^T^ showed 1.14% sequence divergence from *Debaryomyces nepalensis* JCM 2095 and and *Debaryomyces coudertii* JCM 2387, and 0.83% sequence divergence from *Debaryomyces hansenii* NRRL Y-7426. Strain CIG-23H^T^ can utilize more carbon sources than closely related species. The findings suggest that strain CIG-23H^T^ is a novel species of the genus *Debaryomyces*, and we propose to name it as *Debaryomyces apis* f.a., sp. nov. The holotype is CBS 16297^T^, and the isotypes are MTCC 12914^T^ and KCTC 37024^T^. The MycoBank number of *Debaryomyces apis* f.a., sp. nov*.* is MB836065. Additionally, a method using cresol red and Bromothymol blue pH indicator dyes was developed to screen for lipase producers, which is more sensitive and efficient than the currently used phenol red and rhodamine B dye-based screening methods, and avoids the problem of less differentiable zone of hydrolysis.

## Introduction

Insects are highly diverse and abundant organisms that possess remarkable adaptability to various ecological niches. This adaptability is partly attributed to their symbiotic associations with microorganisms that colonize their gut through food, playing a vital role in their health, survival species, and biomass^1^. These microorganisms are acquired through food and are vital to the host’s survival^2^. The insect’s gut has a remarkable diversity of yeasts and bacteria that aid in metabolism, digestion of recalcitrant food products, and protection from pathogens and parasites^1,3^. However, the diversity of yeasts in natural habitats and hosts is often underestimated, particularly in insect gastrointestinal tracts, and further research is needed to uncover their ecological significance^4^. Yeasts have been reported to be primarily associated with insects belonging to the *Coleoptera*, *Hymenoptera*, *Hemiptera*, *Neuroptera*, *Lepidoptera*, *Isoptera*, *Blattodea*, and *Diptera* orders^5^. The discovery of novel yeast species associated with different insect groups can reveal new insights into their mutual associations, role in the insect gut, and phylogenetic relationships. A considerable number of yeast communities are related to the order Hymenoptera, which includes bumblebees, honeybees, wasps, and stingless bees^6–8^. Studies on the symbiosis of different yeast species have been conducted among bees and bee-pollinating plants, which have shown the association of *Candida*, *Cryptococcus*, *Metschnikowia*, *Starmerella*, *Zygosaccharomyces*, and *Debaryomyces* clades with the bees^9,10^. The genus *Debaryomyces* Lodder and kreger-Van Rij was defined by Q-9 coenzyme as major ubiquinone, inability to assimilate nitrate, multilateral budding, and conjugation between bud and cell leads to an ascus formation^11^. Species of the genus *Debaryomyces* were isolated from various sources, such as soil, air, insects, plants, feces, fruits, tree exudates, pollen, seawater, and guts of vertebrates^12,13^.

In this study, new yeast species that are associated with the gut of insects were isolated, and their potential for producing lipase was investigated. Lipases are widely used in various industries, such as food, dairy, detergents, and pharmaceuticals, which has resulted in the continued search for new microorganisms capable of producing thermolipases^14^. Screening for lipase producers is typically performed using various dyes, including phenol red and rhodamine B plate assays. However, rhodamine B plate assay has shown inconsistent results, and phenol red dye produces a less differentiable zone of hydrolysis in screening other important enzymes^15–17^.

Based on their 18S rRNA gene, D1/D2 domain sequences, it was determined that four yeast strains isolated from the insect gut belonged to a novel yeast species for which the name *Debaryomyces apis* f.a., sp. nov., has been proposed. One of the four strains, CIG-23H^T^, was selected for further biochemical and physiological studies. In addition to the isolation process, a new screening method for lipase producers was developed using pH indicator dyes, specifically Bromothymol blue (BTB) and cresol red. This modified method addressed the limitations of previous screening methods that used phenol red and rhodamine B and produced more contrasting and easily distinguishable hydrolysis zones when pH decreased from neutral to acidic.

## Materials and methods

### Isolation of yeast species

The insect sample was collected from the Churdhar, Himachal Pradesh, India (GPS coordinates: 30° 52′ 34.68″ N 77° 24′ 4.68″ E) in June 2017, and abbreviated as CIG (Churdhar Insect Gut). The bees (Hymenoptera order) were collected during the daytime from the forest of Churdhar and kept in a clean, sterile 50 mL screw-capped tube lined with damp filter paper for two days before dissection. Starvation aids in the removal of contaminant species that may have been isolated from the gut^18^. Yeasts were isolated from the insect gut using a previously described method^19^. The gut contents were homogenized and plated on YM agar plates (0.3% yeast extract, 0.5% peptone, 0.3% malt extract, 1% glucose and 2% agar; pH 6.2), PDA plates (20% potatoes infusion form, 2% dextrose, and 2% agar; pH 5.6), and YEPD agar plates (2% peptone, 1% yeast extract, 2% dextrose, and 2% agar) supplemented with 50 mg/L chloramphenicol, and 30 mg/L streptomycin, to inhibit bacterial growth. The selected isolates were purified and preserved at − 80 °C in 15% glycerol and liquid nitrogen^20^.

### Molecular identification by DNA sequencing and sequence analysis

Yeast strains were grown on the YM agar plate at 25 °C and harvested after 48 h of growth. Genomic DNA was extracted using ZR Fungal/Bacterial DNA Miniprep, due to its effectiveness in extracting DNA from tough-to-lyse fungi and yeasts. Each PCR reaction was carried out with a final volume of 50 µL, containing 100–200 ng of genomic DNA, 10 pmol of each primer, 200 µM dCTP, dGTP, dTTP, and dATP, 4 mM MgCl_2_, 5 U of Taq polymerase, and 10 µL of 5× GO Taq Flexi buffer. The ITS region was amplified using ITS1-ITS4 and ITSF-ITS4 primers, while the D1/D2 domain was amplified using NL1 and NL4 primers^21^. Alternatively, the entire ITS region and the D1/D2 domain were amplified using ITS1/ITSF and NL4 primers, and the amplified products were sequenced using the same sets of primers. The 18S rRNA gene sequence was amplified using NS1 and NS8 primers and sequenced by using NS1, NS2, NS3, NS4, NS5, NS6 and NS8. PCR cycling parameters were as follows: initial denaturation for 5 min at 94 °C, followed by 30 cycles of 30 s at 94 °C, 30 s at 55 °C and 1 min (for ITS and D1/D2) and 2 min (for 18S rRNA gene) at 72 °C, followed by 10 min extension at 72 °C, and cooling to 4 °C as a final step^20^. Agarose gel of 1% (w/v) was used for the separation of amplified product and visualized under Fluro Chem E (Cell Biosciences, USA). The amplified region was purified using RBC HiYield Gel/PCR DNA Mini Kit and sequenced with an ABI 313 genetic analyzer (Applied Biosystems, California, USA). The GenBank nBLAST (Basic Local Alignment Sequencing Tool) was used to perform a sequence-similarity search, and the ITS region, D1/D2 domain, and 18S rRNA gene sequences were submitted to the GenBank database. Type strain sequences of closely related species were retrieved from the GenBank database and aligned using CLUSTAL W (version 1.6) in MEGA7^22^. Maximum-likelihood and Neighbor-joining trees were constructed using the concatenated gene sequence of the 18S rRNA and D1/D2 domains of 26S rRNA, and the Kimura two-parameter correction model was used to calculate the evolutionary distance^22–24^. Gaps and missing data were removed from all positions, and the confidence level of the clades was calculated using 1000 iterations of bootstrap values and are expressed as percentages at the nodes^25^. *Schizosaccharomyces pombe* NRRL Y-12796^T^ was used as an out-group.

### Biochemical and morphological characterization

Phenotypic characterization of the strains was performed using standard methods^26^. Carbon assimilation tests were conducted using MICROLOGSYSTEM_TM 4.2_ Biolog YT microplate, which is known for its simplicity, rapidness, accuracy, and ability to provide valuable information on the metabolic properties of a strain, in addition to species level identification (Biolog, Inc., Hayward, CA). The Biolog YT microplate consists of 94 wells and two control wells, and it is divided into two sections: the top section for oxidation reactions (35 carbon sources), and the lower section for assimilation reactions (59 carbon sources). The oxidation wells contain tetrazolium violet, which changes colorless to purple if substrate is oxidized. Metabolic fingerprints of test strains were generated based on the oxidation and assimilation of the carbon sources. To develop metabolic fingerprints, strain CIG-23H^T^ was grown on Biolog Universal Yeast (BUY) agar and inoculated into a YT microplate (96-well plate) on which all nutrients and biochemicals had already been added (prefilled and dried). The YT microplate was incubated for 24, 48, and 72 h at 25 °C as recommended by the manufacturer. The metabolic pattern was analysed using the MICRO LOG softwareTM 3 using its database^20^.

The nitrogen assimilation tests were carried out in 5 mL liquid media in a test tube using yeast carbon base (YCB) and starved inoculum. The carbon fermentation test was performed using standard methods^26^. The carbon and nitrogen sources were sourced from HiMedia (Mumbai, India), while YNB, YCB, peptone, malt extract, and yeast extract were procured from Difco (Detroit, MI). The results were recorded after incubation at 25 °C for 5–21 days. The formation of ascospores was investigated on YM agar, PDA, corn meal agar (CMA), V8 juice agar, and YCB supplemented with 0.01% (w/v) ammonium sulfate with individual and mixed strains at 25 °C for three weeks. Dalmau plate method using YM agar was used to check the pseudo mycelium formation. Growth of the CIG-23H^T^ strain at different temperatures (4 °C, 15 °C, 25 °C, 30 °C, 37 °C, and 42 °C) was evaluated by streaking on YM agar plates. Cell images were captured using a phase-contrast microscope (Olympus SC180 Tokyo, Japan), and their sizes were determined based on ten independent measurements.

### A qualitative and quantitative method for screening of lipase activity

The present study employed a modified version of the phenol red plate assay to evaluate lipase-producing yeasts^27,28^. The tributyrin medium contains peptone, 5 g/L; CaCl_2_, 1 g/L; yeast extract, 5 g/L; tributyrin, 10 mL/L; agar, 20 g/L; phenol red, 0.1 g/L; and had a pH of 7.0^27,28^. Initially yeasts were cultured on a YM agar for 2 days and then streaked onto a tributyrin plate containing phenol red. Subsequently the plates were incubated at 25 °C for 48 to 72 h, and the appearance of yellow color around the colony was recorded as an indication of lipase activity^29^. As the lipase hydrolyzes tributyrin, it releases free fatty acid that lowers the pH of the plate, leading to a color change from red to yellow^27^.

For quantitative analysis, lipase activity was determined spectrophotometrically by using para nitrophenol palmitate (pNPP) as a substrate. The assay was carried out in accordance with the method well described in the literature, with minor modification^30^. A stock solution of 3 mM of pNPP was dissolved in dimethyl sulfoxide (DMSO), and an equal volume of potassium phosphate buffer of 20 mM (pH 6.8) was added. A quantity of 500 µL of the buffered substrate was added to 500 µL of enzyme extract. After 30 min of incubation at 25 °C, the reaction was stopped by adding 250 µL of sodium bicarbonate (0.1 M). The reaction was centrifuged for 2 min at 12,000 rpm to remove any residues. Para nitrophenol (pNP) release was monitored at 405 nm in a cuvette using spectro star nano BMG Labtech (Ortenberg, Germany). A substrate blank contains water instead of the substrate, and the enzyme blank contains water instead of enzyme solution were used in the assay. One enzymatic unit was referred to the amount of enzyme required to release one µ mole of pNP per min under standard assay conditions^30,31^.

### Investigation of various pH indicator dyes for lipase screening

Five pH indicator dyes, including BTB, cresol red, chlorophenol red, bromocresol purple, phenol red, and Rhodamine B were used to screen lipase producers.

### Testing of pH indicators on the agar plate

Lipase-producing yeasts CIG-23H^T^ and *Pseudozyma antarctica* MTCC 2706^T^ were maintained on YM agar plates. The *Escherichia coli* MTCC 1610^T^ was maintained on nutrient agar (NA) plates. The six different dyes were added separately in the production medium i.e., 0.01% phenol red (w/v), 0.01% BTB (w/v), 0.01% cresol red (w/v), 0.01% bromocresol purple (w/v), 0.01% chlorophenol red (w/v) and 0.01% rhodamine B (w/v). The plates were prepared and inoculated individually with CIG-23H^T^ and *P. antarctica*, then incubated for 48 h at 25 °C. The plates inoculated with *E. coli* were incubated at 37 °C for 48 h. Plates containing CIG-23H^T^ were used as test cultures, whereas plates containing *P. antarctica* were used as positive controls and plates containing *E. coli* were used as negative controls. The uninoculated plates with the substrate and inoculated plates without substrate were taken as control.

### Testing of pH indicators in the liquid medium

CIG-23H^T^, *P. antarctica*, and *E. coli* were inoculated in a 100 mL flask containing medium supplemented separately with six different dyes mentioned above. The flasks inoculated with yeasts and bacteria were incubated at 25 °C and 37 °C for 48 h, respectively, at 180 rpm. An uninoculated sterile medium and medium without substrates (inoculated with test culture) were taken as controls.

## Results and discussion

### Phylogeny of *Debaryomyces apis* f.a., sp. nov.

**Figure 1:**
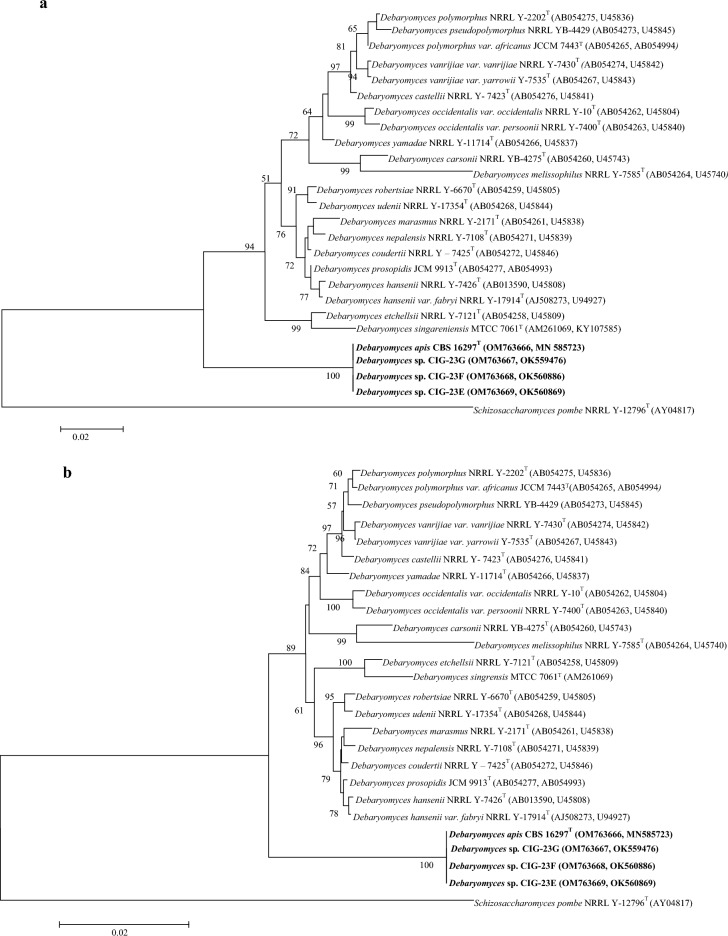
Phylogenetic tree depicting the relatedness of *Debaryomyces apis* sp. nov. CIG-23H^T^ and related species of *Debaryomyces* genus. The maximum-likelihood tree (**a**) and neighbor-Joining tree (**b**) was constructed by using the concatenated gene sequences of the D1/D2 domain and 18S rRNA gene sequence. *Schizosaccharomyces pombe* NRRL Y-12796^T^ was used as an out-group. Bar 0.02 is the substitution per nucleotide position. The bootstrap value greater than 50% are given at nodes.

The analysis of the ITS region, D1/D2 domain, and 18S rRNA gene sequences showed that the strains CIG-23H, CIG-23G, CIG-23E, and CIG-23F were identical. In pairwise sequence alignment of the D1/D2 region (571 bp), CIG-23H^T^ showed sequence divergence of 7.5% from *D. nepalensis* JCM 2095^T^ and 7.8% from *D. udenii* JCM 7855^T^,whereas in the ITS region (579 bp), the strain CIG-23H^T^ showed sequence divergence of 15% from *D. nepalensis* JCM 2095^T^ and 15.3% from *D. subglobosus* JCM 1989^T^ and in the 18S rRNA gene sequence (1637 bp), the strain CIG-23H^T^ showed 1.14% sequence divergence from *D. nepalensis* JCM 2095^T^ and 0.83% from *D. hansenii* NRRL Y-7426^T^ (Table 1). Based on the established guidelines for by Kurtzman and Robnett, identifying ascomycetous yeast species using nucleotide sequence divergences, strains are classified as the same species if they have 0–3 nucleotide differences in the D1/D2 domains. On the other hand, strains that display more than 6 nucleotide differences (equivalent to 1% dissimilarity) in the D1/D2 domains are recognized as different species. Later, Vu et al.^32^ introduced a threshold for taxonomic differentiation, stating that a strain should be classified as a distinct species from its close relatives if it exhibits less than 98.31% similarity (for Ascomycota) or 98.61% similarity (for Basidiomycota) in the ITS region whereas when analyzing the D1/D2 domains, the proposed threshold for species differentiation is set at less than 99.41% similarity (for Ascomycota) or 99.51% similarity (for Basidiomycota). Based on our analysis of the ITS region, D1/D2 domain, and 18S rRNA gene sequences, we observed that our novel isolate exhibited greater than 6% sequence divergence in the D1/D2 domain, 15% in the ITS region, and 1.6% in the 18S rRNA gene compared to the closest type strains. The concatenated sequence of the 18S rRNA gene and D1/D2 domain of the novel strain CIG-23H^T^ and closely related *Debaryomyces* clade members were used to construct a maximum-likelihood (Fig. 1a) and neighbor-joining tree (Fig. 1b). In phylogenetic analysis, the strain CIG-23H^T^ occupied a different branch with strong 94 and 89 bootstrap support. Cletus P. Kurtzman and Motofumi Suzuki^33^ proposed new genera using the sequence of 18S rRNA gene and D1/D2 domain, and classified *Debaryomyces* species into three clades, i.e., *Debaryomyces*, *Schwanniomyces* emend., and *Priceomyces* gen. nov. According to our phylogenetic analysis based on D1/D2 region, the novel strain CIG-23H^T^ falls in the *Debaryomyces* clade.

**Table 1:** Gaps, substitutions, and nucleotide similarity between novel species and its closely related species in the ITS regions, D1/D2 domains, and 18S region.

Number of gaps/nucleotide substitution/nucleotide similarity (%)							
Gene/species	*D. nepalensis* JCM 2095^T^ (NG_055700)	*D. coudertii* JCM 2387^T^ (NG_054827)	*D. udenii* JCM 7855^T^ (NG_054828)	*D. propsopidis* JCM 9913^T^ (NG_055701)	*D. subglobosus* JCM 1989^T^ (NR_077067)	*D. psychrosporus* CBS 11845^T^ (NR_152495)	*D. hansenii* NRRL Y-7426^T^ (NG_063361)
D1/D2	3/45/92.5	3/45/92.5	3/44/92.6	3/41/92	3/43/92.2	–	–
ITS	25/35/85	25/36/84.5	–	–	25/37/84.7	25/35/84.6	–
18S	5/17/98.86	7/27/98.74	–	6/22/98.86	–		5/17/99.17

**Figure 2:**
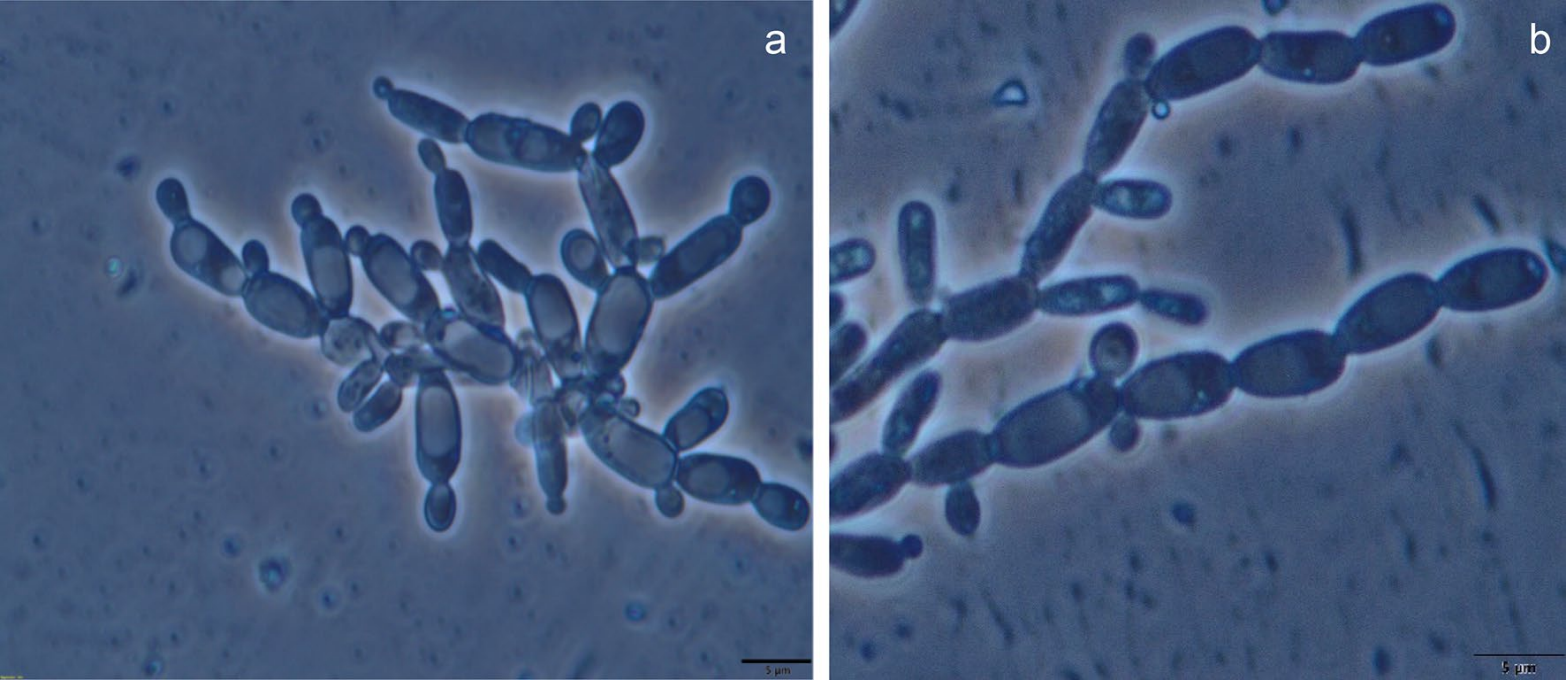
Micrograph of vegetative cells of strain CIG-23H^T^, showing polar budding (**a**) and pseudo hyphae (**b**) formation. The scale bar represents 5 µm.

Colonies of the strain CIG-23H^T^ were raised, glistening, white, entire, and butyrous. Sporulation was not observed even after several attempts on different media after twenty-one days of growth. Polar budding (Fig. 2a) and pseudohyphae (Fig. 2b) were present after the three days of growth on YM agar (Fig. 2). The strain CIG-23H^T^ can be distinguished from their phylogenetically closest recognize neighbor by some phenotypic characters. In the strain CIG-23H^T^, fermentation was very strong and utilized more carbon sources than the closely related species. The strain CIG-23H^T^ can ferment maltose, sucrose, D-trehalose, and assimilate D-ribose, D-arabinose, L-sorbose, and grow in a vitamin-free medium.

The strain CIG-23H^T^ differs from *Debaryomyces singareniensis* in the fermentation of galactose and glucose, assimilation of xylose, L-arabinose, maltose, cellobiose, and salicin. The strain CIG-23H^T^ differs from *Debaryomyces etchellsii* in the fermentation of maltose, galactose, sucrose, d-trehalose, and assimilation of d-arabinose, erythritol, and weak growth in 50% glucose (Table 2). Through phylogenetic analysis of the D1/D2 domain and 18S rRNA gene, along with examination of physiological and biochemical data, we conclude that the CIG-23H^T^ isolate represents a new species within the *Debaryomyces* genus. We therefore propose the name *Debaryomyces apis* f.a., sp. nov.

**Table 2:** Physiological characteristics of *Debaryomyces apis* f.a., sp. nov. CIG-23H^T^ and their closely related type strains.

Biochemical test	MTCC-12914	MTCC-7061*	MTCC-3578*	CBS-8300*
Fermentation of carbon compounds				
Glucose	+	+	+	−
Galactose	+	+	−	−
Maltose	+	−	w/−	−
Sucrose	+	−	w/−	−
d-Trehalose	+	−	−	−
Assimilation of carbon compounds				
d-Ribose	+	w	±	−
d-Xylose	+	−	+	+
l-Arabinose	+	−	+	+
d-Arabinose	w/+	−	−	−
Maltose	+	−	+	−
d-Cellobiose	+	−	+	−
Melezitose	w/+	−	+	−
Salicin	+	−	+	−
Sucrose	w/+	−	+	−
Glycerol	+	+	+	−
l-Sorbose	+	w	+	−
α-α-Trehalose	w/+	−	±	d,w
Erythritol	w/+	+	−	−
Assimilation of nitrogen compounds				
Nitrate	−	−	−	−
d-Glucosamine	+	w	+	−
Lysine	w/+	+	+	−
37 °C	−	−	−	+
50% Glucose	−	−	w	−
60% Glucose	−	−	w	−
DBB test	−	−	−	−
Growth in vitamin-free medium	+	NA	−	−

Physiological traits of type strains 1. *Debaryomyces apis* sp. nov. CIG-23H^T^ (MTCC-12914^T^), 2. *Debaryomyces singareniensis* (MTCC-7061^T^), 3. *Debaryomyces etchellsi* (MTCC-3578^T^), 4. *Debaryomyces mycophilus* (CBS-8300^T^).

*Data from Kurtzman et al*.* The yeasts—A taxonomic study 5th edition 2nd volume (2011).

+  = positive; −  = negative; *w* weak, *d* delayed, *NA* not available, *v* variable.

The description of *Debaryomyces apis* f.a., sp. nov is based on a single strain because multiple strains could not be isolated. Following the description of a species, whether from a single strain or many isolates, additional strains are often recognized from different substrates or geographical areas, resulting in an incremental understanding of the species characteristics, ecology, and distribution^34^. About one-third of all the currently documented yeast species was originally described based on single strains, implying that these species descriptions have considerably contributed to our understanding of the yeast diversity and will continue to do so in the future^35^. Yeast isolated from bees and related insects is reported to hydrolyze lipid (waxes), which is present inside the corolla and nectar^36,37^. The yeast isolated from the beetle’s gut also showed lipolytic activity, suggesting the nutritional process’s role. Lipases produced by the yeasts might help hydrolyze lipids, and energy is used in metamorphosis, dispersion, reproduction, and host colonization^38^. Strain CIG-23H^T^ also showed good lipase activity, which might help in the hydrolysis of lipid and help in insect development. Globally the number of bees is expected to be approximately 20,000, and species from genera *Starmerella*, *Metschnikowia*, and *Debaryomyces* were isolated from bees^7^. Therefore, the number of novel species may rise if we explore the yeast associated with the bees.

### Qualitative and quantitative screening of lipase production from CIG-23H^T^

In the present study, the novel yeast strain CIG-23H^T^ showed hydrolysis zone on tributyrin agar plate supplemented with phenol red. Based on the quantitative screening, the novel yeast strain CIG-23H^T^ showed an enzyme activity of 162 U/mL.

### Evaluation of different pH indicators dyes on the agar plate

**Figure 3:**
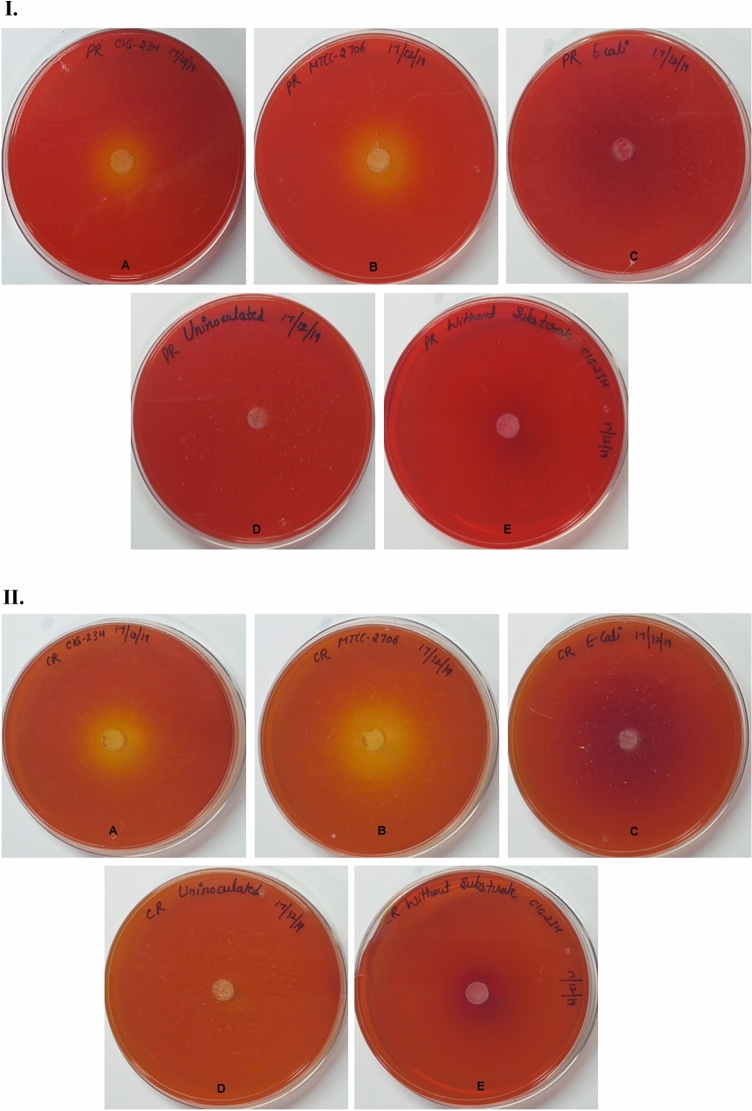

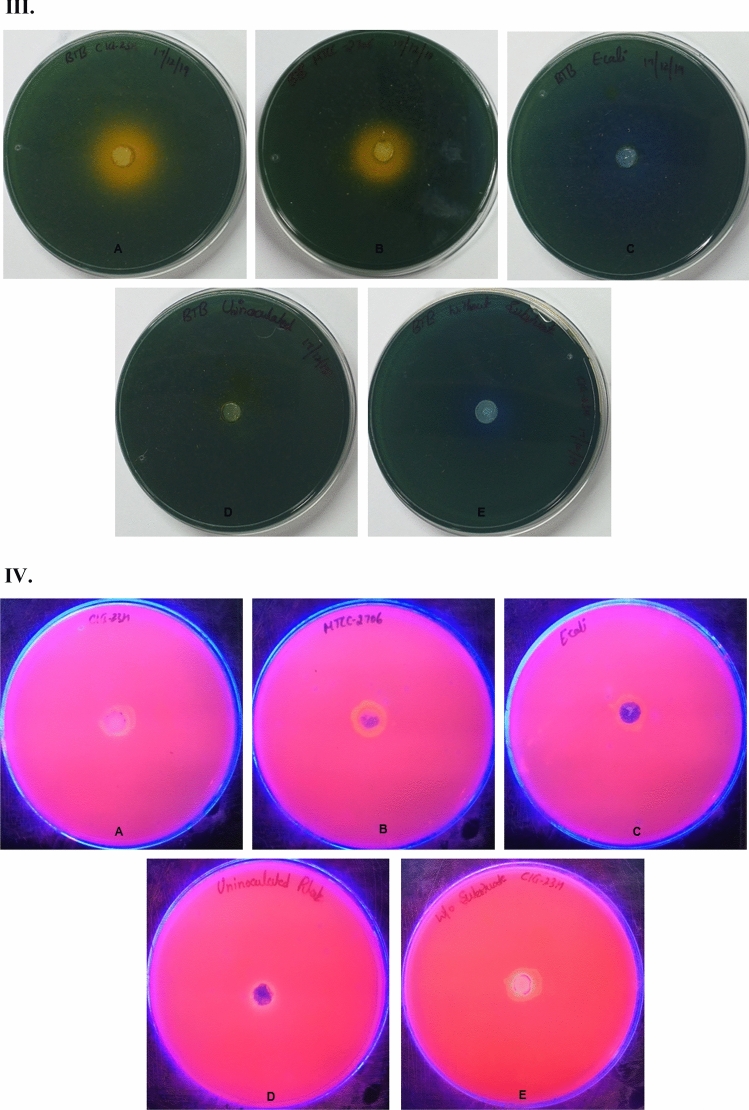

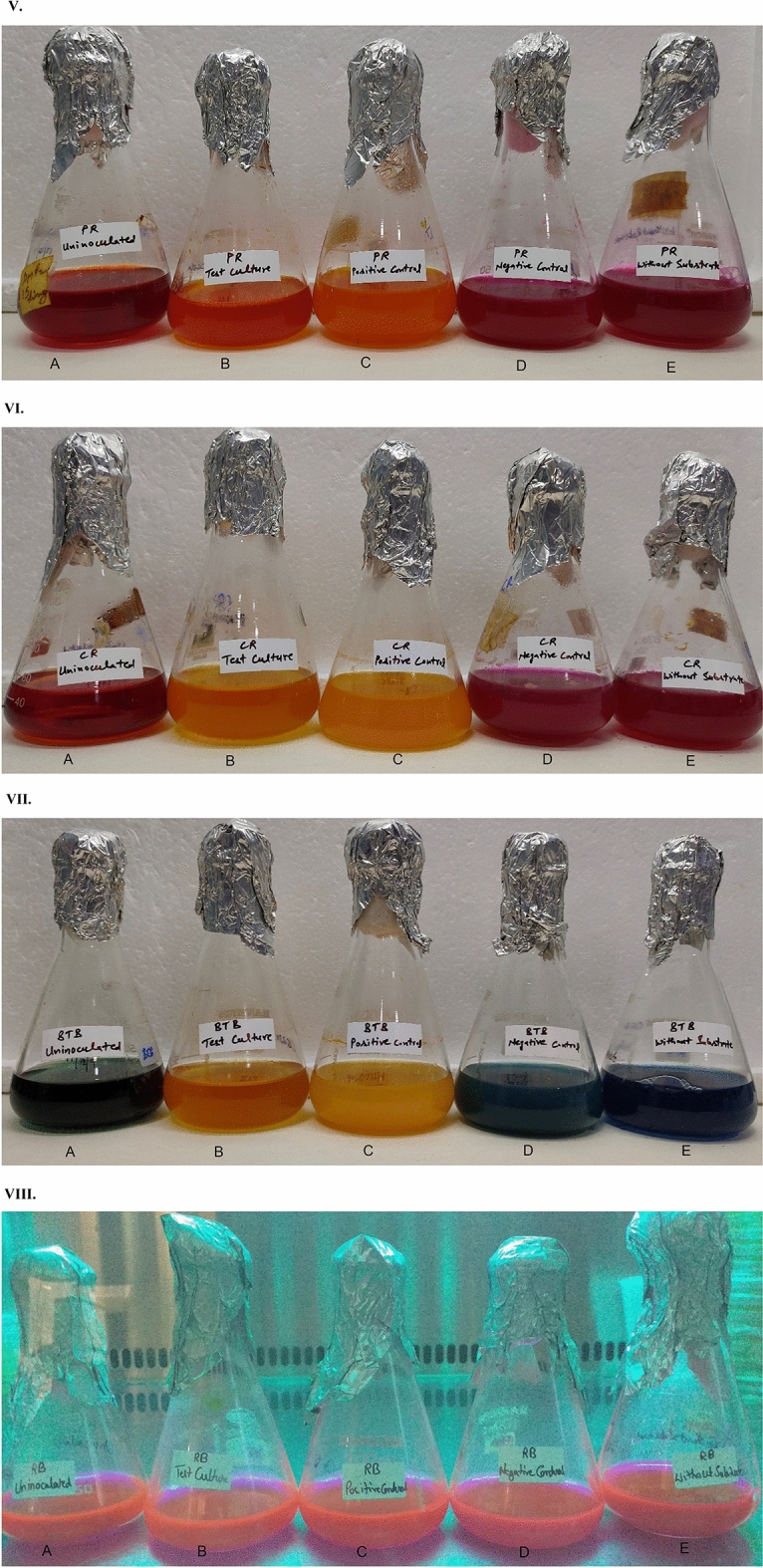
(**I**) Screening of lipase producers on phenol red dye containing plates. (**A**) CIG-23H^T^ (test culture). (**B**) *Pseudozyma antarctica* MTCC 2706 (positive control). (**C**) *E. coli* MTCC 1610 (negative control). (**D**) Uninoculated (control). (**E**) CIG-23H^T^ (without substrate). (**II**) Screening of lipase producers on cresol red dye containing plates. (**A**) CIG-23H^T^ (test culture). (**B**) *Pseudozyma antarctica* MTCC 2706 (positive control). (**C**) *E. coli* MTCC 1610 (negative control). (**D**) Uninoculated (control). (**E**) CIG-23H^T^ (without substrate). (**III**) Screening of lipase producers on BTB dye containing plates. (**A**) CIG-23H^T^ (test culture). (**B**) *Pseudozyma antarctica* MTCC 2706 (positive control). (**C**) *E. coli* MTCC 1610 (negative control). (**D**) Uninoculated (control). (**E**) CIG-23H^T^ (without substrate). (**IV**) Screening of lipase producers on Rhodamine B dye containing plates. (**A**) CIG-23H^T^ (test culture). (**B**) *Pseudozyma antarctica* MTCC 2706 (positive control). (**C**) *E. coli* MTCC 1610 (negative control). (**D**) Uninoculated (control). (**E**) CIG-23H^T^ (without substrate). (**V**) Screening of lipase producers in phenol red dye containing flasks. (**A**) Uninoculated (control). (**B**) CIG-23H^T^ (test culture). (**C**) *Pseudozyma antarctica* MTCC 2706 (positive control). (**D**) *E. coli* MTCC 1610 (negative control). (**E**) CIG-23H^T^ (without substrate). (**VI**) Screening of lipase producers in cresol red dye containing flasks. (**A**) Uninoculated (control). (**B**) CIG-23H^T^ (test culture). (**C**) *Pseudozyma antarctica* MTCC 2706 (positive control). (**D**) *E. coli* MTCC 1610 (negative control). (**E**) CIG-23H^T^ (without substrate). (**VII**) Screening of lipase producers in BTB dye containing flasks. (**A**) Uninoculated (control). (**B**) CIG-23H^T^ (test culture). (**C**) *Pseudozyma antarctica* MTCC 2706 (positive control). (**D**) *E. coli* MTCC 1610 (negative control). (**E**) CIG-23H^T^ (without substrate). (**VIII**) Screening of lipase producers in Rhodamine B dye containing flasks. (**A**) Uninoculated (control). (**B**) CIG-23H^T^ (test culture). (**C**) *Pseudozyma antarctica* MTCC 2706 (positive control). (**D**) *E. coli* MTCC 1610 (negative control). (**E**) CIG-23H^T^ (without substrate).

In the current study, five various pH indicator dyes and Rhodamine B dye were tested to screen lipase-producing microorganisms (Fig. 3). Following 24 h to 48 h incubation, the hydrolysis zone was observed in phenol red plate inoculated with test culture CIG-23H^T^ (Fig. 3IA) and positive control culture *P. antarctica* (Fig. 3IB). The cresol red dye-containing plates showed a contrasting zone of hydrolysis when inoculated with test culture and positive control (Fig. 3IIA,IIB). In the case of medium plates supplemented with BTB, the test culture CIG-23H^T^ and positive control culture *P. antarctica* produced a differentiable zone of hydrolysis (Fig. 3IIIA,IIIB). The cresol red and BTB dye supplemented plates showed a zone of hydrolysis with high intensity, contrast, and differentiable from the unhydrolyzed part of the plate. Whereas phenol red produced zone of hydrolysis with less intensity and differentiability to unhydrolyzed part of the plate in comparison of cresol red and BTB. No color change was observed in plates containing bromocresol purple (SF1), bromocresol green, and chlorophenol red (SF2). In case of Rhodamine B plates, no halos were observed when visualized under UV light at 350 nm after 48 h of incubation (Fig. 3IVA–E). There was no change in color found in uninoculated plates (Fig. 3ID,IID,IIID) while without substrates (inoculated with test culture) showed the color change, which was due to the increase in pH not related to lipase activity (Fig. 3IE,IIE,IIIE). All the plates inoculated separately with negative control also showed an increase in pH of the plate with no lipase activity (Fig. 3IC,IIC,IIIC). From the above results, it could be concluded that cresol red and BTB dyes are better than phenol red for the screening of lipase producers in terms of intensity, contrasting, and differentiable zone of hydrolysis.

### Evaluation of different pH indicators dyes in the liquid medium

The liquid medium in flask was separately supplemented with five pH indicator dyes and Rhodamine B dye and inoculated with CIG-23H^T^, *P. antarctica*, and *E. coli*. The uninoculated flasks and flasks without substrates (inoculated with test culture) were labeled as controls. After incubation, the phenol red dye supplemented flasks showed a change in color with test culture CIG-23H^T^ and *P. antarctica* compared to the control flask (Fig. 3VA–C). In the case of cresol red and BTB supplemented flasks, a tremendous change in color was observed upon inoculation with test culture CIG-23H^T^ and positive control culture *P. antarctica* (Fig. 3VIA–C). In comparison to phenol red, the intensity of color change from neutral to acidic in cresol red and BTB dye was highly distinguishable. The negative control flask and flask without substrates showed a slight increase in pH, unrelated to the lipase activity (Fig. 3VD,E). In the case of Rhodamine B dye, no color change was observed when visualized under UV light at 350 nm after 48 h of incubation (Fig. 3VIIIA–E). The results of the above comparison study showed that the BTB and cresol red dyes worked well in a broth and could be used as potential viable screening method for lipase-producing yeasts.

## Conclusion

In conclusion, the novel strain CIG-23H^T^ *Debaryomyces apis* f.a., sp. nov. showed more than 5% sequence divergence in the ITS and the D1/D2 region. The strain CIG-23H^T^ also showed the biochemical and physiological differences from closely related type species. This data supports the proposal of this strain as a novel yeast species. The novel strain also showed the lipase activity of 162 U/mL and was used as a test culture in the modified screening method. The current report developed a modified pH indicator dye-based method using 2 dyes, i.e., BTB and cresol red, which showed a better contrasting and differentiable zone of hydrolysis. The BTB and cresol red plate assay method illustrated to be easy, efficient, better, and more differentiable than the phenol red and rhodamine B for screening lipase-producing microorganisms.

### Taxonomic description of new species

#### *Debaryomyces apis* f.a., sp. nov.

##### *Debaryomyces apis* (a’pis. L. gen. n. apis of a bee family apidae)

The colonies exhibit a raised, glistening, entire, white, and butyrous appearance after growing on YM agar at 25 °C for 3 days. The cells appear oval and are arranged in chains. The spore formation is not observed on cornmeal and YM agar after 21 days of growth. Polar budding and pseudohyphae are present after the three days of growth on YM agar.

The organism ferments dextrin, d-cellobiose, inulin, gentiobiose, d-melezitose, maltose, d-melibiose, d-mannitol, d-raffinose, palatinose, d-trehalose, d-glucose, d-sorbitol, l-sorbose, sucrose, salicin, and d-galactose, and assimilates d-gluconic acid, maltose, dextrin, inulin, gentiobiose, d-cellobiose, d-raffinose, d-meleziotose (weak), d-melibiose (weak), salicin, sucrose, *N*-acetyl d-glucosamine, d-glucose, d-galactose, l-rhamnose, d-trehalose, d-glucosamine, l-sorbose, d-mannitol, d-sorbitol, d-ribose, d-arabitol, l-arabinose, xylitol (weak), tween 80 (weak), erythritol (weak), d-arabinose (weak), glycerol, d-xylose. However, it does not assimilate d-psicose, α-ketoglutaric acid, maltitol, and α–methyl d glucoside.

The organism assimilates lysine (weak) and cadaverine, but not creatine, creatinine, nitrate, nitrite, and ethylamine. Growth at 12 °C, 25 °C, and 30 °C is positive, but negative at 37 °C and 42 °C. Acid generation from glucose on Custer’s chalk medium is positive, and growth on 10% (w/v) NaCl agar, 10% (w/v) NaCl/5% (w/v) glucose, 16% (w/v) NaCl agar, 16% (w/v) NaCl/5% (w/v) glucose, 50% (w/v) glucose and 60% (w/v) glucose is negative. The organism does not grow in 1% (v/v) acetic acid medium, and its starch-like compound formation and gelatin liquefication are negative. The diazonium blue B (DBB) reaction and urea hydrolysis are negative, and growth in vitamin free medium is positive.

The holotype of *Debaryomyces apis* is strain CBS 16297^T^_,_ deposited in the CBS collection of the Westerdijk Fungal Biodiversity Institute, Utrecht, The Netherlands. The isotypes, MTCC 12914 = KCTC 37024 were deposited in a metabolically inactive state in the Microbial Type Culture Collection and Gene Bank (MTCC), Chandigarh, India, and Korean Research Institute of Bioscience and Biotechnology. The MycoBank number of *Debaryomyces apis* f.a., sp. nov., is MB836065.

The datasets presented in this study can be found in NCBI under the accession number MN585723 (D1/D2 domain of CIG-23H), OK559476 (D1/D2 domain of CIG-23G), OK560886 (D1/D2 domain of CIG-23F), OK560869 (D1/D2 domain of CIG-23E), OM763666 (18S rRNA gene of CIG-23H), OM763667 (18S rRNA gene of CIG-23G), OM763668 (18S rRNA gene of CIG-23F), and OM763669 (18S rRNA gene of CIG-23E).

## Supplementary Information

Supplementary Figures.

## Data Availability

This published article contains all the data generated or analyzed during this study. The datasets presented in this study can be found in online depositories. Depositories: the strain CIG-23H^T^ has been deposited in CBS (CBS 16297^T^), KCTC (KCTC 37024^T^), MTCC (MTCC 12914^T^), and in NCBI under the accession number MN585723 (D1/D2 domain of CIG-23H), OK559476 (D1/D2 domain of CIG-23G), OK560886 (D1/D2 domain of CIG-23F), OK560869 (D1/D2 domain of CIG-23E), OM763666 (18S rRNA gene of CIG-23H), OM763667 (18S rRNA gene of CIG-23G), OM763668 (18S rRNA gene of CIG-23F), and OM763669 (18S rRNA gene of CIG-23E).
