# Supplementary material for: Description of lipase producing novel yeast species Debaryomyces apis f.a., sp. nov. and a modified pH indicator dye-based method for the screening of lipase producing microorganisms
Source: Sci Rep. 2023 Jul 21;13:11819. doi: 10.1038/s41598-023-38241-3 (PMC10362016; doi:10.1038/s41598-023-38241-3)

Research Article

Description of lipase producing novel yeast genus *Churdharomyces apis* gen. nov., f.a., sp. nov. and a modified pH indicator dye-based method for the screening of lipase producing microorganisms

Alka Kumari[1], Kanti N. Mihooliya[2], Debendra K. Sahoo[2], Mani S. Bhattacharyya[2], Gandham S. Prasad[1#*], Anil K. Pinnaka[1*]

Supplementary Figure SF1 Screening of lipase producers on bromocresol purple dye containing plates.

(A) CIG-23H T (test culture)

(B) *Pseudozyma antarctica* MTCC 2706 (positive control)

(C) *E. coli* MTCC 1610 (negative control)

(D) CIG-23H T (without substrate)

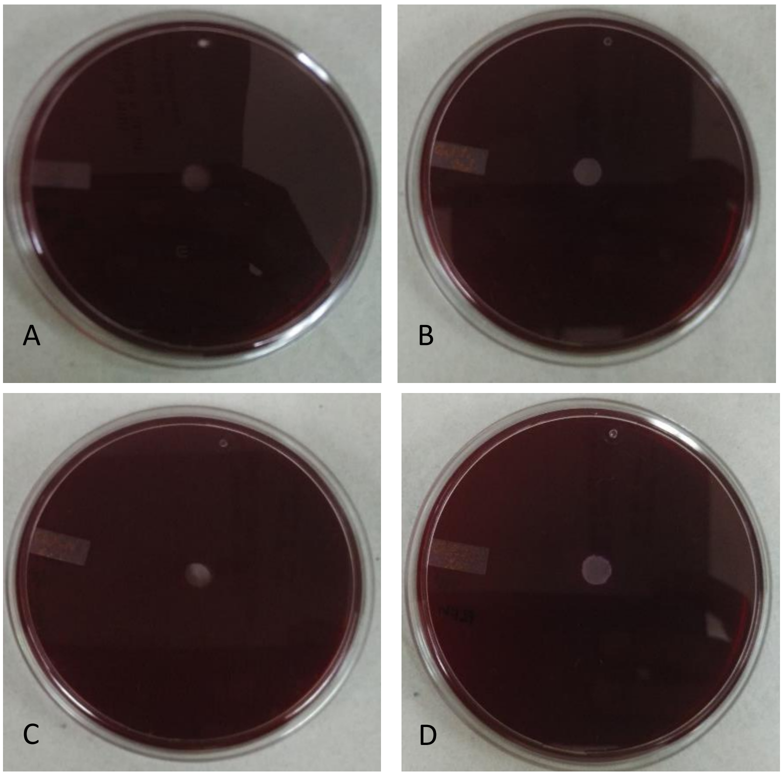

Supplementary Figure SF2 Screening of lipase producers on chlorophenol red dye containing plates.

(A) CIG-23H T (test culture)

(B) *Pseudozyma antarctica* MTCC 2706 (positive control)

(C) *E. coli* MTCC 1610 (negative control)

(D) CIG-23H T (without substrate)

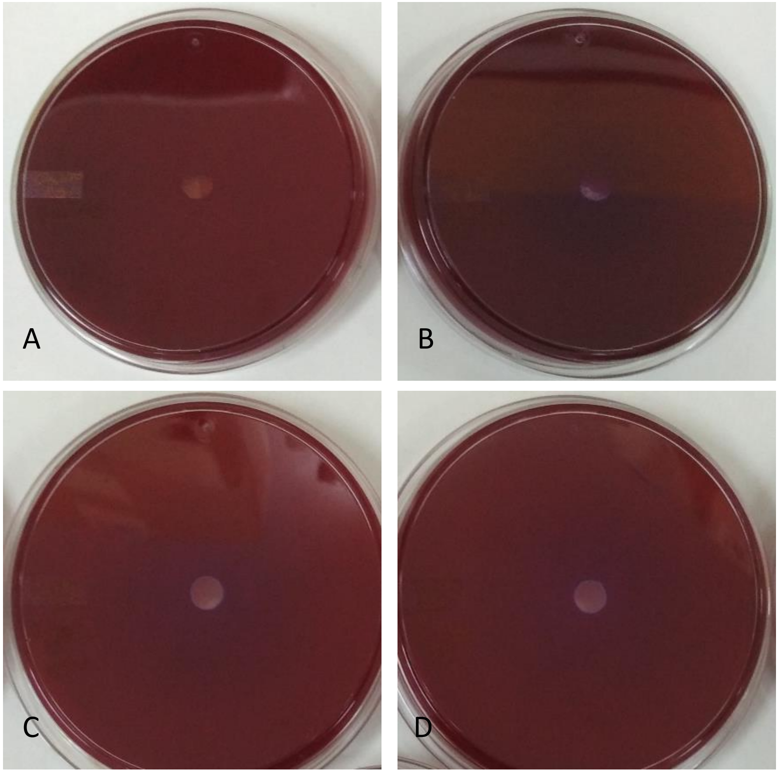

Supplement: Supplementary file 1 — Supplementary Figures. [file 41598_2023_38241_MOESM1_ESM.pdf]
